# Natural Bioactive Agents as Adjunctive Candidates in Periodontitis: Mechanistic Links to Cardiovascular Disease and Translational Challenges

**DOI:** 10.3390/biomedicines14102324

**Published:** 2026-10-13

**Authors:** Claudia Reytor-González, Martín Campuzano-Donoso, Dolores Jima Gavilanes, Raquel Horowitz, Daniel Simancas-Racines

**Affiliations:** 1 Center for Evidence Ecosystems, Implementation Science, Decision-Making (CIDES), Facultad de Ciencias de la Salud y Bienestar Humano, Universidad Tecnológica Indoamérica, Ambato 180150, Ecuador; martincd01@hotmail.com; 2 Escuela de Medicina, Universidad Espíritu Santo, Samborondón 0901952, Ecuador; 3 Valley Health System, Geriatric Medicine, Glen Rock, NJ 07452, USA

**Keywords:** periodontitis, cardiovascular disease, natural bioactive agents, polyphenols, systemic inflammation, drug delivery systems, host modulation, translational medicine

## Abstract

Periodontitis is a highly prevalent chronic inflammatory disease that destroys the tooth-supporting tissues and has been consistently associated, in observational studies, with low-grade systemic inflammation and atherosclerotic cardiovascular disease, although whether this reflects a direct contribution to vascular disease, shared risk factors, or both remains unresolved. Subgingival instrumentation is the standard of care. Natural bioactive agents have been investigated as adjuncts because selected formulations exhibit antimicrobial, anti-inflammatory, antioxidant, pro-resolving, or reparative actions. This critical narrative review analyzes mechanistic, preclinical, and clinical evidence and asks whether effects on pathways shared by periodontal and vascular biology constitute a plausible, but unproven, link to cardiovascular health. Clinical trials have reported small, short-term changes in periodontal surrogate outcomes for selected formulations. They do not establish non-inferiority or equivalence to chlorhexidine, and none assessed cardiovascular events. Local gels, nanoparticles, and adhesive local formulations are being developed to address the poor bioavailability limiting several agents, though clinical validation remains preliminary. Formulations may improve periodontal exposure, but they add a second experimental variable and remain incompletely validated in humans. Systemically administered omega-3 fatty acids, melatonin, vitamin D, and polyphenols also require separate consideration because direct extra-periodontal effects and drug interactions can occur independently of periodontal change. Natural bioactive agents should therefore be regarded as investigational adjuncts rather than replacements for mechanical therapy. Their cardiovascular relevance remains a testable translational hypothesis that requires route-specific pharmacokinetics, human target-engagement evidence, adequately powered periodontal trials, and eventual cardiovascular outcome studies.

## 1. Introduction

Severe periodontitis had an estimated global age-standardized prevalence of 12.5% in 2021, corresponding to approximately 1.07 billion cases, and the number of cases is projected to increase by 44.3% by 2050 [1]. Its clinical consequences extend well beyond tooth loss and impaired masticatory function. Large real-world cohorts encompassing close to 100,000 patients show measurable associations with cardiovascular disease, cancer, and all-cause mortality, at a scale that reframes periodontitis as a public health problem rather than a purely dental one [2]. The disease is not simply a local bacterial infection but a disorder in which a dysbiotic subgingival biofilm triggers a dysregulated host inflammatory response that destroys alveolar bone and periodontal ligament while allowing cytokines, endotoxin, and bacteria to enter the systemic circulation [3]. This distinction, between periodontitis as a passive site of infection and periodontitis as a potential contributor to chronic low-grade systemic inflammation, underlies the oral-systemic health paradigm [4].

Cardiovascular disease remains the leading cause of death globally, and epidemiological, microbiological, and mechanistic evidence has associated periodontitis with atherosclerotic risk, although the independence and causal nature of this relationship remain uncertain. The Joint European Federation of Periodontology/American Academy of Periodontology workshop on periodontitis and systemic diseases formally acknowledged this association while cautioning that observational evidence, however consistent, does not by itself establish causation [5]. Shared risk factors such as smoking, diabetes, and aging complicate this picture, and residual confounding cannot be excluded in most of the cohort and case–control literature; nonetheless, the recurrence of pathogen dissemination, systemic cytokine elevation, endothelial dysfunction, and prothrombotic changes across independent lines of evidence has kept periodontitis on the list of candidate cardiovascular risk factors first proposed over two decades ago [6]; this remains an unresolved, rather than an established, hypothesis.

Subgingival instrumentation, together with effective supragingival biofilm control and supportive care, remains the cornerstone of periodontal therapy. The EFP S3 clinical practice guideline does not support routine adjunctive use of antiseptics or systemic antibiotics for most patients. These measures are reserved for selected indications after benefit-risk assessment [7,8]. Chlorhexidine can cause tooth staining, taste disturbance, and mucosal irritation, while antimicrobials raise resistance concerns. Natural products are therefore considered potential adjuncts within evidence-based care, not substitutes for mechanical treatment or responses to a presumed universal need for chlorhexidine. Mechanical therapy can control the infectious and inflammatory burden but has limited capacity to reconstruct the original architecture and function of tissues already destroyed. Regenerative biomaterials are relevant to this distinct therapeutic goal because cytocompatible and bioactive materials can interact with the tissue environment and support reconstruction after infection and inflammation are controlled [9]. Host modulation and regeneration should not be conflated.

Natural bioactive agents have emerged within this host-modulation paradigm as candidates of particular interest, not because they are inherently superior to synthetic drugs, but because selected agents act on pathways implicated in periodontal destruction and vascular biology. Polyphenols, flavonoids, terpenoid-related compounds, alkaloids, and characterized natural preparations have been reported to inhibit nuclear factor kappa B and the NOD-like receptor family pyrin domain containing 3 (NLRP3) inflammasome, activate the nuclear factor erythroid 2-related factor 2/heme oxygenase-1 antioxidant program, influence the receptor activator of nuclear factor kappa-B ligand/osteoprotegerin axis, or disrupt periodontopathogenic biofilms [10]. These mechanisms remain distinct from clinical efficacy. Local subgingival delivery, topical oral use, and systemic administration are analyzed separately because exposure, retention, safety, and the possibility of direct extra-periodontal effects differ by route. Comparative evidence remains too heterogeneous to establish class-wide equivalence to chlorhexidine [11].

Existing evidence on natural bioactive agents in periodontitis is heavily weighted toward preclinical models and short-term surrogate endpoints, is undermined by considerable heterogeneity in formulation, dosing, and outcome definitions, and has yet to be tested in trials that combine periodontal and cardiovascular endpoints within the same design [12].

This review therefore pursues three linked objectives: first, to critically analyze the epidemiological, mechanistic, and interventional evidence linking periodontitis with systemic inflammation and cardiovascular disease, while examining the limitations of causal inference; second, to examine the mechanistic rationale for natural bioactive agents as candidates capable of acting on inflammatory pathways proposed to be shared by periodontal and vascular tissue; and third, to critically analyze the nature, consistency, methodological limitations, and translational maturity of the experimental, preclinical, and clinical literature supporting their use as adjunctive periodontal therapies. To connect rather than juxtapose these aims, shared pathways are treated as candidate targets, after which each agent is interpreted by administration route, human periodontal evidence, safety, and the evidentiary steps required before any cardiovascular inference.

## 2. Methodology

Given the clinical and methodological heterogeneity of the evidence, and the objective of integrating periodontal, cardiovascular, mechanistic, and translational literature, a critical narrative synthesis was considered most appropriate. A structured literature search of PubMed/MEDLINE and Europe PMC was conducted from database inception through September 2026. Search concepts combined terms related to periodontitis, cardiovascular disease and atherosclerosis, community dysbiosis, the NLRP3 inflammasome, oral nitrate reduction, causal inference and Mendelian randomization, natural bioactive agents and individual candidate compounds, regenerative biomaterials, local delivery systems, safety, and natural product–drug interactions.

Priority was given to current clinical practice guidelines, systematic reviews and meta-analyses, randomized clinical trials, large observational studies, and human pharmacokinetic or safety studies. Mechanistic in vitro and animal evidence was included when it addressed a pathway or translational question not adequately examined in humans. Such evidence was used to evaluate biological plausibility and was not interpreted as evidence of human clinical efficacy. Reference lists of relevant publications were checked manually to identify additional sources.

Because this article was designed as a narrative review, no protocol was registered. PRISMA reporting, exhaustive systematic screening, duplicate screening, independent duplicate data extraction, formal study-level risk-of-bias assessment, certainty grading, and de novo quantitative synthesis were not performed. Publications were selected purposively according to relevance, study design, methodological rigor, recency, and their capacity to support, qualify, or challenge the claims examined. Findings were integrated thematically rather than pooled quantitatively.

Evidence was interpreted according to design and inferential strength. In vitro experiments were treated as evidence of molecular plausibility, animal studies as preclinical evidence subject to model-specific limitations, and observational studies as human associations that remain vulnerable to confounding. Periodontal clinical outcomes, pathway-specific target engagement, vascular intermediate outcomes, and cardiovascular events were treated as distinct outcome levels. Local subgingival delivery, topical oral exposure, and systemic administration were also evaluated separately because formulation, exposure, safety, and possible extra-periodontal effects differ by route.

A targeted supplemental literature check addressed the current burden of periodontitis, contemporary dysbiosis models, inflammasome biology, periodontal-systemic mechanisms, metabolic effect modification, oral nitrate reduction, evidence for and against vascular colonization, Mendelian randomization, periodontal standards of care, regenerative biomaterials, experimental delivery systems, regional publication bias, agent-specific safety, and interactions with cardiovascular medications. Bibliographic records were verified against PubMed, Europe PMC, Crossref, or official journal records.

## 3. From Local Dysbiosis to Systemic Inflammation: Rethinking Periodontitis

### 3.1. Beyond Bacterial Infection: The Host Response as the Primary Driver of Periodontal Destruction

Periodontitis begins with a community-level ecological shift rather than domination by a fixed list of red-complex organisms. Inflammation changes nutrient availability and the local environment, favoring inflammophilic and immunogenic communities that provide a broad range of pathogen-associated molecular patterns [13]. The contribution of *Aggregatibacter actinomycetemcomitans* varies by phenotype and population and should not be generalized to all periodontitis. *Porphyromonas gingivalis* remains relevant as a possible keystone organism because even at low abundance it can disrupt the balance between innate immune surveillance and microbial tolerance [14]. Toll-like receptors recognize microbial products and activate nuclear factor kappa B and signal transducer and activator of transcription 3, driving transcription of interleukin-1 beta, interleukin-6, tumor necrosis factor alpha, and interleukin-17 [15]. Salivary lipopolysaccharide also shows chemically and biologically heterogeneous properties across periodontal states [16]. Disease expression therefore reflects interactions among the microbial community, its functional products, host susceptibility, and a dysregulated inflammatory response.

Inflammasomes are intracellular multiprotein complexes that activate inflammatory caspases, permit maturation and release of interleukin-1 beta and interleukin-18, and can induce pyroptosis [17]. In periodontitis, signals from a dysbiotic subgingival community may prime and activate the NLRP3 inflammasome in host cells. Canonical and non-canonical pyroptotic pathways have been observed in experimental periodontal lesions, and their inhibition reduces cytokine release and alveolar bone loss in animal models [18]. Receptor-interacting protein kinase 3-dependent necroptosis in gingival fibroblasts has likewise been shown to worsen experimental periodontitis [19]. Excessive inflammasome activity could therefore maintain a local environment that is unfavorable to inflammatory resolution and tissue repair. This is a preclinical hypothesis. Suppressing NLRP3 alone does not reconstruct cementum, periodontal ligament, or alveolar bone, and no human trial has demonstrated periodontal regeneration through inflammasome modulation.

### 3.2. Molecular Networks Underlying Periodontal Disease

The transcriptional and signaling architecture of periodontitis converges on a limited number of hub pathways, a feature that offers a rationale for how single, multi-target agents might plausibly modulate a disease that superficially appears combinatorially complex; whether this rationale translates into clinical benefit is addressed in later sections. NF-κB functions as a central transcriptional regulator: it integrates Toll-like receptor, cytokine receptor, and oxidative stress signals to drive transcription of pro-inflammatory mediators and matrix metalloproteinases, and its chronic activation in gingival tissue correlates with disease severity in human biopsy studies [14]. The mitogen-activated protein kinase (MAPK) cascade operates in parallel, amplifying cytokine production and directly promoting osteoclast differentiation, such that p38 MAPK inhibition has been proposed as an adjunctive strategy for limiting bone loss [20]. Genome-wide and bioinformatic analyses of periodontal tissue have additionally identified regulatory hubs beyond these canonical pathways, though their relevance to the natural agents discussed later in this review has not been specifically established and is not developed further here [21].

Bone destruction in periodontitis is mediated principally through the receptor activator of nuclear factor kappa-B ligand (RANKL) and its decoy receptor osteoprotegerin (OPG): periodontal pathogens and pro-inflammatory cytokines shift this ratio toward RANKL, promoting osteoclast differentiation and alveolar bone resorption, while bacterial lipopolysaccharide directly upregulates matrix metalloproteinase and tissue inhibitor expression in gingival fibroblasts, degrading the connective tissue matrix that would otherwise support the alveolar bone [22]. Oxidative stress compounds this destructive program at every level: reactive oxygen species generated by activated neutrophils and by dysregulated lipid metabolism not only damage periodontal tissue directly but also activate c-Jun N-terminal kinase and NF-κB in a feed-forward loop that has been demonstrated experimentally to propagate injury from periodontal tissue to distant organs such as the liver [23]. Neutrophils deserve particular attention in this network because persistent low-grade endotoxemia reprograms myelopoiesis itself, generating a population of hyper-responsive neutrophils prone to excessive neutrophil extracellular trap (NET) formation; these NETs, while important for pathogen containment, also cause collateral tissue injury and, when their clearance is impaired, sustain a self-perpetuating cycle of local and systemic inflammation [13].

### 3.3. Systemic Consequences of Periodontal Inflammation

The periodontal pocket is not a sealed compartment. Ulcerated pocket epithelium, which, in a patient with generalized periodontitis, can present a surface area comparable to the palm of a hand, provides a chronic portal through which bacteria, bacterial products, and inflammatory mediators enter the systemic circulation during ordinary function such as chewing and brushing [24]. Transient bacteremia is well-documented; endotoxemia represents an additional proposed route: circulating lipopolysaccharide and other pathogen-associated molecular patterns engage Toll-like receptors on hepatocytes, adipocytes, and vascular endothelial cells, activating the same NF-κB/STAT3 circuitry locally responsible for periodontal destruction, now in distant organs [25]. Experimental oral administration of *P. gingivalis* has also been shown to induce measurable dysbiosis of the gut microbiota and impaired intestinal barrier function in animal models, one proposed route by which oral dysbiosis could plausibly extend to hepatic and metabolic disease, though this oral–gut–liver pathway has not been established in humans and is not developed further in this review [26]. Circulating pro-inflammatory cytokines, chief among them IL-1β, IL-6, and TNF-α, together with elevated C-reactive protein, constitute the biochemical signature of this systemic spillover and are consistently elevated in patients with moderate-to-severe periodontitis independent of other cardiometabolic risk factors [3]. Oxidized low-density lipoprotein, elevated both locally and systemically in periodontitis, further links periodontal inflammation to lipid metabolism, promoting neutrophil extracellular trap formation and providing one of several proposed routes toward atherogenic vascular injury [27,28].

Two features of this evidence base warrant explicit caution. First, most mechanistic evidence derives from cell culture or animal models, particularly ligature-induced periodontitis in rodents. The ligature model is not purely mechanical because it promotes plaque accumulation and a host inflammatory response, but it creates an acute perturbation over days or weeks and does not reproduce the natural history, immune adaptation, or comorbidity profile of human periodontitis developing over years. Supraphysiological concentrations used in vitro add a separate exposure problem. Second, much of the pathway literature is correlative. Elevated nuclear factor kappa B activity, oxidative stress, or regulated cell death may be causal drivers, downstream consequences, or both. These models are valuable for hypothesis generation and target prioritization, but they cannot establish human therapeutic efficacy or cardiovascular benefit.

## 4. The Oral-Cardiovascular Axis: Current Evidence and Biological Plausibility

### 4.1. Epidemiological Evidence Linking Periodontitis and Cardiovascular Disease

The epidemiological case for an association between periodontitis and cardiovascular disease is now built on a substantial body of longitudinal evidence rather than isolated case reports. A ten-year retrospective cohort demonstrated that patients with severe periodontal disease had significantly elevated incidence of acute myocardial infarction and stroke compared with periodontally healthy controls, an association that persisted after adjustment for smoking, diabetes, and hypertension [29]. Behavioral proxies for periodontal health corroborate this signal from a different angle: infrequent tooth brushing is associated with higher cardiovascular event rates in a large national survey cohort, an association that, as with the cohort evidence above, cannot exclude unmeasured behavioral, socioeconomic, and health-engagement confounding, which likely accounts for at least part of the signal [30]. A systematic review and meta-analysis extends this pattern to tooth loss, which independently predicts cardiovascular mortality across heterogeneous populations [31]. Importantly, the association is not confined to older or comorbid populations: an analysis of the National Health and Nutrition Examination Survey spanning 1999–2014 found a significant link between periodontitis and atherosclerotic cardiovascular disease among non-Hispanic White adults without other overt risk enrichment, which somewhat narrows, though does not eliminate, the plausible role of shared behavioral or socioeconomic confounding [32].

### 4.2. Shared Molecular and Cellular Mechanisms

Biological plausibility for the epidemiological association rests on several proposed mechanisms. Polymerase chain reaction studies have detected periodontal bacterial DNA in thrombi from patients with acute myocardial infarction [33]. This demonstrates the presence of genetic material but not bacterial viability, persistent endothelial colonization, direction of spread, or causation. Controlled human studies have also reported no detection of target periodontal species in carotid specimens [34] or no recovery by culture despite occasional polymerase chain reaction signals [35]. In contrast, a small proof-of-concept study reported viable invasive *A. actinomycetemcomitans* and *P. gingivalis* in atherosclerotic plaque [36]. These discordant, predominantly cross-sectional findings support episodic vascular exposure to oral microbial material, but they do not establish stable colonization or a causal contribution to human atherogenesis.

A multifaceted framework proposed by Romandini and colleagues organizes periodontal-systemic links into seven domains: microbial translocation, systemic inflammation, maladaptive myelopoiesis, immune-cell trafficking, masticatory and dietary consequences, functional microbiome dysregulation, and shared susceptibility or exposures [37]. This framework connects mechanisms that might otherwise appear as separate narratives, while remaining conceptual rather than proof that any domain increases cardiovascular events.

Host metabolic context may amplify these processes. Experimental evidence reviewed by Preshaw and colleagues indicates that periodontitis alone can produce systemic effects in some models, but responses are often more pronounced with diabetes, obesity, or related metabolic dysregulation [38]. This supports prespecified interaction testing in human studies; it does not establish cardiovascular effect modification.

The enterosalivary nitrate–nitrite–nitric oxide pathway provides an additional functional hypothesis. Oral bacteria reduce dietary nitrate to nitrite, which can contribute to nitric oxide generation and vascular homeostasis [39]. A small human study found reduced *ex vivo* oral nitrate-reduction capacity in periodontitis and improvement after periodontal treatment, while basal plasma nitrate and nitrite did not change [40]. No vascular function or cardiovascular event was measured. The pathway is therefore emerging and biologically plausible, not established.

### 4.3. Causality, Controversies, and Current Limitations

**Figure 1:**
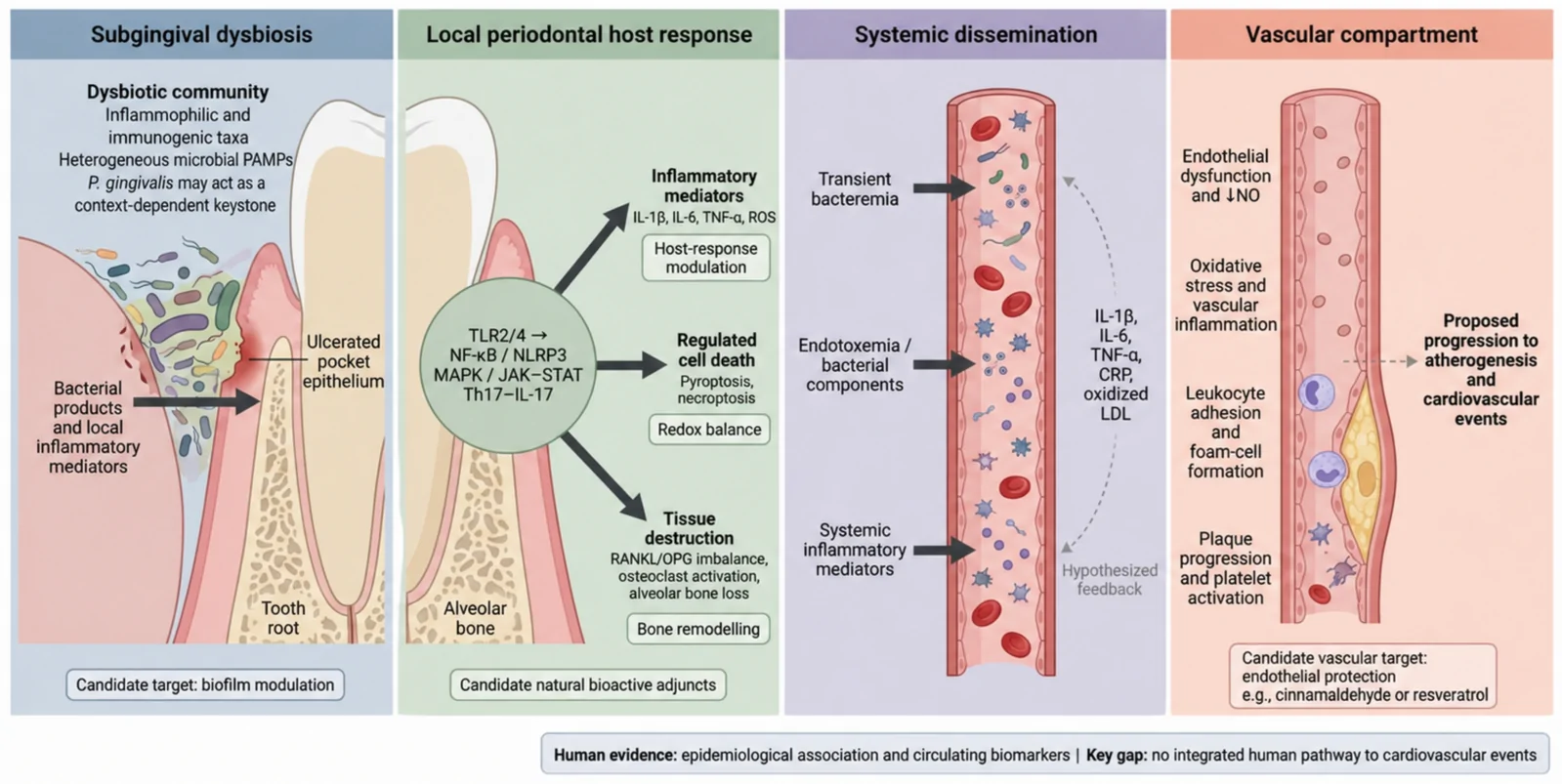
Proposed biological links between periodontitis and cardiovascular disease. Subgingival dysbiosis and ulceration of the periodontal pocket may initiate a local host response characterized by inflammatory signaling, regulated cell death, oxidative stress, connective-tissue degradation, and alveolar bone loss. Bacteremia, bacterial components, and circulating inflammatory mediators provide plausible routes through which periodontal inflammation may extend beyond the oral compartment and contribute to vascular dysfunction. In the vascular compartment, endothelial dysfunction, reduced nitric oxide bioavailability, oxidative stress, leukocyte adhesion, foam-cell formation, and plaque progression have been reported in experimental and observational studies. Candidate natural bioactive adjuncts are positioned at periodontal and vascular intervention points for which supporting evidence has been reported. The link from periodontitis to atherogenesis and cardiovascular events is shown as biologically plausible and epidemiologically associated, rather than as a demonstrated causal pathway. [3,10,13,14,15,16,17,18,19,24,25,26,27,28,33,34,35,36,37,38,39,40] Abbreviations: TLR, Toll-like receptor; NF-κB, nuclear factor kappa B; NLRP3, NOD-like receptor family pyrin domain containing 3; MAPK, mitogen-activated protein kinase; JAK/STAT, Janus kinase/signal transducer and activator of transcription; Th17, T-helper 17; IL, interleukin; TNF-α, tumor necrosis factor alpha; ROS, reactive oxygen species; NO, nitric oxide; CRP, C-reactive protein; LDL, low-density lipoprotein; RANKL, receptor activator of nuclear factor kappa-B ligand; OPG, osteoprotegerin.

Mendelian randomization provides a complementary test of causality but depends on the strength and specificity of the available genetic instruments. Bell and colleagues reported null estimates per doubling of genetically predicted periodontitis odds for any stroke (odds ratio, 0.99; 95% confidence interval, 0.97 to 1.02), ischemic stroke (1.00; 95% confidence interval, 0.97 to 1.03), coronary artery disease (1.01; 95% confidence interval, 0.99 to 1.03), and carotid intima-media thickness (beta = −0.002; 95% confidence interval, −0.004 to 0.001) [41]. Ma and colleagues likewise found no association with overall ischemic stroke (odds ratio, 1.017; 95% confidence interval, 0.992 to 1.043), although an exploratory cardioembolic-stroke estimate was borderline (1.052; 95% confidence interval, 1.002 to 1.104) and relied on a relaxed instrument threshold [42]. These results do not support a clear causal effect. Periodontal treatment can reduce inflammatory biomarkers and improve arterial stiffness, but these are surrogate outcomes [43]. No convincing evidence shows that treatment prevents myocardial infarction, stroke, or cardiovascular death, and reporting spin in this literature warrants continued caution [12]. These proposed periodontal–vascular pathways and potential intervention points for natural bioactive adjuncts are summarized in Figure 1, highlighting their biological plausibility without implying established cardiovascular causality.

## 5. Natural Bioactive Agents as Multi-Target Adjunctive Candidates

### 5.1. Defining Natural Bioactive Agents

The mechanistic account developed in Section 3 and Section 4 identifies candidate pathways that may be relevant in both periodontal and vascular tissues. A shared pathway is not itself a therapeutic bridge. For that designation, an agent would need plausible route-specific exposure, demonstrable target engagement in humans, an associated periodontal benefit, and evidence that any vascular signal is mediated by periodontal improvement rather than a direct systemic action. Natural bioactive agents are of interest as low-affinity, multi-target candidates, but chemical heterogeneity and broad activity also increase uncertainty about selectivity and mechanism [10].

### 5.2. The Rationale and Limitations of Multi-Target Pharmacology

This multi-target profile should not be read as an unqualified advantage. Low-affinity activity across several targets can reflect poor selectivity and a greater risk of off-target effects. For most agents, concentrations required for a given in vitro effect have not been shown to be achievable in periodontal tissue at tolerated doses. Comparative trials against chlorhexidine were not designed with prespecified equivalence or non-inferiority margins. Failure to detect a statistically significant difference in small superiority trials does not demonstrate therapeutic parity [11]. Therapeutic equivalence is therefore not established.

### 5.3. Host-Modulatory Potential

The potential clinical relevance of this multi-target profile should be considered within current standards of care. Mechanical instrumentation remains primary, while chlorhexidine and antibiotics are selective rather than universal adjuncts [7,8]. A small gingivitis trial reported fewer local adverse effects with one herbal mouthwash than with chlorhexidine [44], but this cannot be generalized to periodontitis or to other agent classes. Omega-3 fatty acids act as precursors of specialized pro-resolving mediators, whereas melatonin, vitamin D, and polyphenols have different antioxidant, endocrine, or immunomodulatory rationales [45]. This mechanistic diversity supports agent-specific investigation rather than a single class-wide therapeutic claim.

### 5.4. Challenges in Therapeutic Development

The same multi-target, low-affinity pharmacology that gives natural agents their mechanistic breadth is also the source of their principal translational liabilities. Several isolated phytochemicals, particularly curcumin and resveratrol, have low systemic oral bioavailability, a consequence of low aqueous solubility, rapid first-pass or salivary metabolism, and poor membrane permeability, limitations well documented in the broader oncology chemoprevention literature and only indirectly extrapolated to the periodontal context [46]; locally administered formulations face a distinct set of challenges related to solubility, stability, pocket retention, salivary clearance, and tissue penetration, rather than systemic bioavailability per se. Extraction methodology further compounds this problem: solvent choice, temperature, and extraction time materially alter the yield and bioactivity of a given plant extract, so that two products both labeled "curcumin extract" or "green tea catechins" may differ substantially in their active constituent profile, undermining reproducibility across studies and, ultimately, across patients [47]. This lack of standardization extends to formulation and dosing: unlike chlorhexidine, whose concentration and application protocol are pharmacopeially fixed, most natural agents are tested at heterogeneous doses, in heterogeneous vehicles (gels, rinses, chips, nanoemulsions), and for heterogeneous durations across the available trial literature, a methodological patchwork that limits meta-analytic pooling and regulatory clarity alike [10]. Long-term safety and efficacy data remain correspondingly sparse; most trials of natural periodontal adjuncts run six months or less, leaving the durability of clinical benefit, and any late-emerging adverse effects, essentially untested [48]. Finally, regulatory pathways for natural products differ substantially by jurisdiction and rarely map cleanly onto the evidentiary standards applied to conventional pharmaceuticals, creating genuine uncertainty about how a promising natural bioactive agent would actually be brought to registered clinical use even if a well-designed pivotal trial were to demonstrate efficacy [49].

Taken together, these considerations position natural bioactive agents as mechanistically rational adjunctive candidates whose principal barriers to translation are pharmaceutical, methodological, and regulatory as much as biological. These findings support further investigation of selected agents as adjunctive candidates while precluding their characterization as established alternatives.

## 6. Evidence by Agent Class and Administration Route

The agents discussed here are chemically, pharmacologically, and biologically heterogeneous. The term natural bioactive agent is used operationally and does not imply a shared mechanism or risk profile. The core synthesis is limited to non-living compounds or sufficiently characterized preparations used as adjuncts to nonsurgical periodontal care. Site-directed subgingival formulations, topical oral preparations, and systemically administered agents are interpreted separately. Hyaluronic acid is classified as a locally delivered reparative biomaterial, and vitamin D supplementation in deficient participants is interpreted as correction of a systemic deficiency. Live probiotics are outside the pharmacological core.

### 6.1. Isolated Natural-Product-Derived Molecules and Botanical Extracts

Polyphenols. Curcumin, the principal curcuminoid of *Curcuma longa*, has the largest clinical literature in this field. A meta-analysis of 23 trials comparing locally delivered curcumin or turmeric plus scaling and root planing with scaling and root planing alone reported an additional probing-depth reduction of 0.47 mm (mean difference, −0.47 mm; 95% confidence interval, −0.88 to −0.06; I^2^ = 95.5%) and a clinical-attachment difference of −0.33 mm (95% confidence interval, −0.54 to −0.11; I^2^ = 62.3%) [50]. A later synthesis of 25 randomized trials produced similar estimates but again reported high heterogeneity [51]. A direct comparison of curcumin with chlorhexidine included 26 trials and 1266 participants. Chlorhexidine was favored for probing depth and attachment loss, 24 trials were rated at high risk of bias, and no study used a prespecified non-inferiority or equivalence margin [52]. Curcumin may have a small formulation-specific adjunctive effect, but a standardized regimen and therapeutic equivalence to chlorhexidine have not been established.

Resveratrol, a stilbene polyphenol concentrated in grape skin and red wine, has been evaluated through systemic and topical routes. A 2026 meta-analysis of seven randomized trials involving 402 participants reported pooled differences favoring resveratrol for probing depth (−0.42 mm; 95% confidence interval, −0.59 to −0.25; I^2^ = 99%) and clinical attachment level (−0.36 mm; 95% confidence interval, −0.52 to −0.19; I^2^ = 98%) [53]. Extreme heterogeneity in route, dose, population, and follow-up prevents inference about a reproducible regimen. No periodontal resveratrol trial assessed cardiovascular outcomes, and any direct systemic effect would need to be separated from mediation through periodontal improvement.

Green tea catechins, principally epigallocatechin gallate, inhibit periodontal pathogen growth and biofilm formation in vitro [54]. A meta-analysis of four small trials reported an additional probing-depth reduction of 0.74 mm (95% confidence interval, 0.35 to 1.13) with locally delivered green tea plus scaling and root planing [55]. The limited number of studies, short follow-up, and differences among gels, strips, rinses, and dentifrices reduce confidence and preclude treating these routes as interchangeable. A propolis review identified six studies, with only three suitable for pooling. Mean probing-depth reduction was reported as 1.49 mm at 30 to 45 days and 0.80 mm at 90 days, but all studies were at high risk of bias and certainty was very low [56]. Separate clinical literature suggests effects of ingested propolis on inflammatory biomarkers [57], but this does not demonstrate cardiovascular benefit from subgingival propolis.

Grape-seed extract illustrates the need to separate human periodontal data from integrated animal findings. One small randomized study allocated 86 sites from five patients and found no between-group probing-depth benefit [58]. A separate 72-patient trial reported greater probing-depth and relative-attachment improvements at three months, but its indexed report did not provide between-group effect sizes or confidence intervals [59]. In a ligation model, grape-seed extract reduced experimental atherosclerosis alongside periodontitis [60]. The latter is integrated preclinical evidence, not human cardiovascular evidence. Other flavonoids, isoflavones, and isolated small molecules remain predominantly at the mechanistic or preclinical stage and cannot support class-wide clinical claims.

No dedicated randomized periodontal trial of isolated isoflavones such as genistein or daidzein was identified. Mechanistic plausibility by chemical-class analogy does not establish periodontal efficacy, and correction of a nutritional or endocrine deficiency should not be equated with intrinsic periodontal pharmacology.

Alkaloids. Alkaloids are represented in the periodontal literature chiefly through berberine and through traditional Chinese medicine formulations containing alkaloid-rich botanicals. Multi-omics and network pharmacology analyses of these formulations have identified immunomodulatory effects attributable in part to alkaloid constituents, though these studies characterize whole-formulation effects rather than isolated alkaloid pharmacology, limiting mechanistic attribution to any single compound [61]; the Kouqiangjie formula has been similarly dissected to identify pharmacodynamic substances acting on inflammatory and microbial targets relevant to periodontitis [62]. This class remains the least characterized at the single-molecule level: no periodontal clinical trial of an isolated alkaloid was identified, and cardiovascular relevance has not been examined in the periodontal literature reviewed here.

Terpenoids. Terpenoids, a group that includes essential oil constituents, ursolic acid, ginsenosides, platycodin D, carvacrol, hinokitiol, and glycyrrhizin, form a chemically diverse class unified by antimicrobial and anti-inflammatory activity. A systematic review of preclinical animal studies concluded that terpenes exert reproducible anti-inflammatory and osteoprotective effects in experimental periodontitis, but the same review explicitly noted that evidence for this class relies almost entirely on animal models, with a corresponding scarcity of human clinical data [63]. Ursolic acid specifically inhibits the RIPK3/NLRP3 signaling axis in experimental periodontitis [64]. Hinokitiol and dipotassium glycyrrhizate enhance macrophage efferocytosis in experimental models [65]. Overall, terpenoid-related agents remain predominantly preclinical: their periodontal effects have been examined mainly in animal or cellular models, and their vascular relevance, where proposed, derives from separate experimental systems. Clinical translation is currently limited by the absence of standardized formulations, human pharmacokinetic data, and periodontal randomized trials.

Other natural-product-derived small molecules. Cinnamaldehyde, a phenylpropanoid structurally distinct from the terpenoid class, was shown in a murine model to restore endothelial function disrupted by *P. gingivalis*, acting through the PPARγ/Akt/eNOS and Nrf2/ARE signaling axes; this vascular finding remains to be confirmed in humans and has no corresponding periodontal efficacy data of its own [66].

Natural peptides. Bioactive peptides, comprising marine- and plant-derived antimicrobial peptides, represent the least clinically mature class reviewed here. A comprehensive review of marine bioactive substances against periodontitis identified antimicrobial peptides as possessing antibacterial and anti-inflammatory activity relevant to periodontal pathogens, noting that clinical validation for this class lags substantially behind the preclinical evidence [67]; the same literature also describes phlorotannins and sulfated polysaccharides, polyphenolic and carbohydrate-based structures grouped here because they were evaluated in the same source studies rather than because they share a chemical class with peptides. No dedicated randomized trial of an isolated natural peptide for periodontitis was identified, and no standardized formulation or human pharmacokinetic data exist for this class.

Polyherbal botanical extracts. Botanical and polyherbal preparations, including Morus alba extracts and Triphala, a multi-herb formulation, show antimicrobial activity against *gingivalis* in vitro that approaches chlorhexidine, alongside antioxidant and anti-inflammatory effects, though clinical trials of these preparations remain few, small, and geographically concentrated in South and Southeast Asia; the degree to which the extracts used across these studies were chemically characterized to a common standard is not consistently reported, so "standardized" should not be assumed for this group as a whole. Polyherbal mouthwash formulations, which combine several of the individual agents discussed above, have been evaluated in single- and double-blind randomized trials, with one topical polyherbal phytopreparation demonstrating measurable reductions in gingival inflammation supported by in silico target prediction analysis; however, no trial identified formally tested for synergy, and head-to-head comparisons between polyherbal and single-extract formulations remain too few to determine whether combination products offer any interaction beyond the additive effect of their individual components [68]. Overall, polyherbal extracts remain predominantly at the small-trial stage: formulation composition is inconsistently reported, chemical standardization cannot be assumed, and geographic concentration of the available trials limits generalizability.

### 6.2. Route-Specific Interpretation of Endogenous and Dietary Agents

Administration route is central to interpreting the endogenous and dietary agents considered here. Systemic omega-3 fatty acids, melatonin, and vitamin D may act outside the periodontal compartment, so a vascular or metabolic signal cannot automatically be attributed to improved periodontal inflammation. Hyaluronic acid is different: it is a locally applied reparative biomaterial with no established independent cardiometabolic evidence.

Omega-3 fatty acids are precursors of specialized pro-resolving mediators. A 2026 review of 16 randomized trials reported additional improvements at three months in clinical attachment (−0.49 mm; 95% confidence interval, −0.75 to −0.23) and probing depth (−0.44 mm; 95% confidence interval, −0.62 to −0.25), with similarly small six-month estimates and high heterogeneity [69]. Periodontal studies used variable eicosapentaenoic and docosahexaenoic acid exposures and sometimes low-dose aspirin. Their findings do not establish a periodontal dose or show that independent cardiovascular effects are mediated by periodontal improvement. A 2026 melatonin meta-analysis included 18 trials and 828 participants. Clinical attachment favored melatonin at two, three, and six months, while probing depth favored it at two and three months but not six months; prediction intervals crossed the null [70]. Vitamin D evidence is more limited. In a 27-participant double-blind pilot trial restricted to patients with serum 25-hydroxyvitamin D below 30 ng/mL, supplementation produced no significant between-group periodontal difference [71]. This finding concerns deficiency correction and cannot establish a pharmacological effect in vitamin-D-replete patients. Topical hyaluronic acid was associated with small pooled probing-depth and attachment differences, but prediction intervals crossed the null and no cardiovascular outcome was assessed [72].

### 6.3. Agents Outside the Core Pharmacological Scope

Probiotics are live biotherapeutics whose effects depend on strain identity, viability, ecological interactions, and a distinct regulatory framework [73]. They are therefore excluded from the core pharmacological synthesis and are not used to support class-wide claims for natural bioactive compounds.

### 6.4. Differences in Translational Development Across Agent Classes

This landscape does not resolve into a simple hierarchy. Curcumin has the largest local-adjunct evidence base, whereas resveratrol, omega-3 fatty acids, and melatonin include systemic or mixed routes. Hyaluronic acid is a local reparative biomaterial, vitamin D evidence concerns deficiency correction, and probiotics are excluded from the pharmacological core. Translational maturity and safety must therefore be assessed agent by agent and route by route rather than inferred from the umbrella category.

## 7. Cross-Cutting Mechanisms: Periodontal and Cardiovascular Evidence Compared

This section synthesizes the available evidence on mechanistic pathways shared across agent classes, distinguishing whether each pathway has been documented in periodontal tissue, vascular tissue, integrated models encompassing both compartments or human studies. The resulting cross-cutting comparison is summarized in Table 1.

NF-κB. In periodontal tissue, NF-κB activation downstream of Toll-like receptor engagement drives IL-1β, IL-6, and TNF-α transcription and consequent bone loss, inhibited in vitro and in animal models by curcumin, resveratrol, and polyphenols broadly, at points including receptor engagement, IκB kinase activity, and nuclear translocation [10,74]. In the vasculature, the same transcription factor, activated by periodontal pathogens or their components detected in systemic or vascular compartments, has been shown in separate experimental systems to upregulate endothelial adhesion molecules and promote monocyte recruitment into the nascent atherosclerotic plaque [75]. Direct evidence that natural-agent NF-κB inhibition demonstrated in periodontal models also occurs in vascular tissue exposed to periodontal pathogens remains largely preclinical and has not been tested in an integrated model.

NLRP3 inflammasome. Inhibition of NLRP3 by natural agents such as ursolic acid reduces pyroptosis and alveolar bone resorption in periodontal tissue [64]. NLRP3 is also implicated in experimental atherosclerosis, where its inhibition has been mechanistically linked to reduced vascular macrophage pyroptosis and plaque instability, although this evidence derives from a separate cardiovascular literature rather than from studies using periodontal-pathogen exposure [76].

Nrf2/KEAP1. In periodontal experimental models, curcumin, resveratrol, and EGCG have been reported to activate Nrf2-dependent antioxidant responses and reduce reactive oxygen species [77]. In vascular endothelial models exposed to periodontal pathogens, Nrf2-related signaling has been associated with preservation of nitric oxide bioavailability, a determinant of vascular tone; this was tested in a vascular experimental model using periodontal-pathogen exposure in one study, in which nitric oxide and Nrf2 signaling jointly protected human umbilical vein endothelial cells against dysfunction induced by periodontal pathogens [78].

RANKL/OPG. Curcumin, resveratrol, propolis, and vitamin D downregulate RANKL and/or support OPG expression in periodontal tissue, limiting alveolar bone loss [10]. This pathway’s cardiovascular relevance is less direct than NF-κB or Nrf2 and rests on a separate literature examining osteoprotegerin as a marker, and possibly mediator, of vascular calcification and endothelial apoptosis; one in vitro study found that osteoprotegerin itself protects endothelial cells against apoptotic death induced by *P. gingivalis* cysteine proteinases, an association best interpreted as mechanistically suggestive rather than adjudicated, given the limited periodontal-specific human evidence on this connection [79].

MAPK, JAK/STAT, and Notch. These pathways are each modulated by natural agents in periodontal tissue, with less direct evidence connecting periodontal and cardiovascular manifestations. MAPK inhibition by flavonoids reduces cytokine production and osteoclastogenesis in periodontal tissue; the compounds shown to inhibit MAPK in periodontal contexts (baicalin, EGCG) have been tested for vascular MAPK inhibition chiefly in non-periodontal cardiovascular pharmacology literature rather than in integrated oral-vascular models [80]. JAK/STAT inhibition by curcumin and resveratrol reduces immune dysregulation locally in periodontal tissue; JAK/STAT signaling has separately been implicated in endothelial inflammation and atherosclerosis progression in a non-periodontal cardiovascular literature [81]. The Notch pathway, modulated by flavonoids and curcumin, regulates osteocyte survival and macrophage polarization in periodontal tissue and, independently, endothelial progenitor cell activity during vascular repair in a non-periodontal cardiovascular literature [82,83]. In both cases the periodontal and vascular findings come from parallel rather than jointly designed studies.

Biofilm disruption. Polyphenols and terpenoids disrupt *P. gingivalis* biofilm architecture and quorum sensing in vitro, an effect demonstrated for EGCG and various essential oil constituents [54]. This mechanism is relevant to cardiovascular biology only indirectly, through its potential to reduce the periodontal pathogen burden available for systemic dissemination; whether it measurably lowers the bacteremic contribution to atherosclerotic plaque formation in humans has not been tested directly.

Endothelial protection. Restoration of nitric oxide bioavailability, reduction of NADPH oxidase-derived reactive oxygen species, and direct anti-adhesive effects on the vascular wall constitute a pathway supported by a vascular experimental model using periodontal-pathogen exposure: cinnamaldehyde restored PPARγ/Akt/eNOS and Nrf2/ARE signaling in endothelial cells exposed to periodontal pathogens, an experimental design built to test the periodontal-pathogen-to-vascular-endothelium link rather than to characterize integrated periodontal and cardiovascular disease jointly [66].

Collectively, these findings indicate that nuclear factor kappa B, NLRP3, Nrf2/KEAP1, and endothelial signaling have evidence on both sides of the oral-vascular axis, but the periodontal and vascular findings were usually generated in separate systems. The grape-seed study is an uncommon integrated animal model [60]. Such designs remain preclinical and do not establish that a periodontal adjunct engages the same target in humans or changes vascular risk.

Candidate biomarkers must be interpreted according to their specificity. C-reactive protein and interleukin-6 do not demonstrate nuclear factor kappa B inhibition, oxidative-stress markers do not directly establish Nrf2 activation, and interleukin-1 beta is not specific to NLRP3. The human periodontal trials reviewed here generally did not measure pathway-proximal targets in gingival tissue or crevicular fluid. Observed changes in probing depth or attachment therefore cannot be attributed to the molecular mechanisms described in vitro. Future studies should prespecify target-engagement assays and mediation analyses before using these pathways to support vascular inference.

## 8. Delivery Technologies as an Additional Experimental Variable

**Figure 2:**
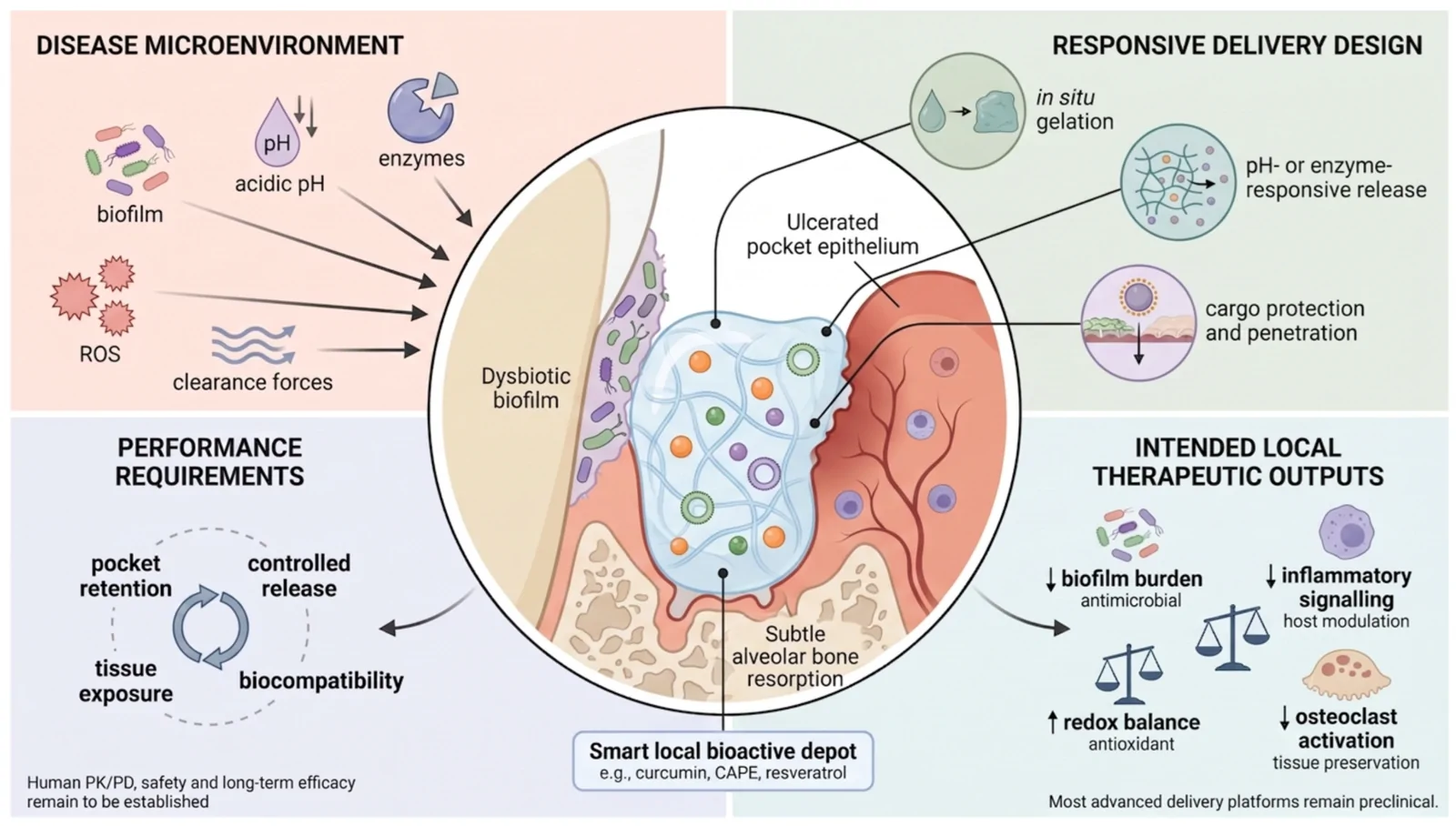
Smart local delivery of natural bioactive agents in the periodontal pocket: microenvironment-responsive design and therapeutic opportunities. The periodontal pocket presents a hostile delivery environment characterized by dysbiotic biofilm, acidic pH, host- and bacteria-derived enzymes, reactive oxygen species, crevicular-fluid flow, and salivary or mechanical clearance. Responsive hydrogels, mucoadhesive polymers, nanoparticles, and vesicle-based systems are designed to retain and protect natural bioactive agents locally and to release them at sites of active inflammation. The intended local outputs are reduced biofilm burden, modulation of host inflammatory signaling, restoration of redox balance, and support of periodontal tissue preservation; these platforms remain predominantly preclinical, add a carrier as a second experimental variable, and require separate human pharmacokinetic, safety, and efficacy validation. [10,84,85,86,87,88,89,90,91,92] Abbreviations: ROS, reactive oxygen species; pH, hydrogen ion concentration; CAPE, caffeic acid phenethyl ester; PK/PD, pharmacokinetic/pharmacodynamic. Symbols: ↑, increase or improvement; ↓, decrease or reduction; solid arrows, direction of proposed processes or effects; circular arrows, interrelated performance requirements; dashed lines, conceptual relationships rather than established causal associations.

The pharmacokinetic limitations identified in Section 5, Section 6 and Section 7, including poor solubility, salivary clearance, and instability in the periodontal pocket, motivate investigation of local delivery systems [10]. These systems are not a validated translational bridge. A hydrogel, polymer, nanoparticle, or vesicle is a second experimental variable with its own composition, release kinetics, tissue exposure, toxicity, manufacturability, and regulatory requirements. Any benefit must therefore be attributed separately to the active agent, the carrier, or their interaction. The periodontal pocket microenvironment and the proposed responsive local delivery strategies are illustrated in Figure 2.

Stimuli-responsive hydrogels currently represent the most extensively developed delivery platform. Thermosensitive hydrogels undergo a sol-to-gel phase transition at body temperature, allowing a natural agent to be injected as a free-flowing liquid into the periodontal pocket and then form a semi-solid depot in situ, minimizing invasiveness while prolonging local retention; an acetylated carboxymethyl chitosan hydrogel loaded with caffeic acid phenethyl ester exemplifies this approach, evaluated in a rodent periodontitis model, where it produced sustained local release together with reduced alveolar bone loss and inflammatory markers, a preclinical rather than clinical stage of development [84]. pH-responsive hydrogels exploit the acidic microenvironment characteristic of active periodontal inflammation, using protonation-dependent swelling or deswelling of polymer networks to trigger drug release preferentially at sites of ongoing disease activity; this targeting strategy has been characterized at the in vitro release-kinetics stage, with periodontal outcome and safety data in vivo still limited [85]. Enzyme-responsive systems extend this logic further, exploiting bacterial or host proteases enriched at infected sites to cleave linkers and release drug specifically where microbial or inflammatory activity is highest; this approach likewise remains at the proof-of-concept, in vitro or early animal stage [86]. Multifunctional hydrogels integrating antibacterial, immunomodulatory, and osteogenic activity within a single construct have also been explored in animal models, including one combining periodontal and systemic comorbidity endpoints, though this work remains preclinical and has not extended to human safety or efficacy data [87].

Natural polymer-based mucoadhesive systems, built from chitosan, alginate, natural gums, and cellulose derivatives, address a complementary problem: retention against salivary flow and mechanical clearance during mastication and oral hygiene. These polymers enhance mucoadhesion and, in some formulations, enable more controlled drug release, while offering intrinsic biocompatibility and biodegradability; the available evidence for this platform is predominantly at the in vitro release-characterization stage, without dedicated periodontal clinical outcome data [88]. Nanoparticle-based platforms, the third major pillar, are designed to improve tissue penetration, protect labile compounds such as curcumin and resveratrol from premature degradation, and enable surface modification for more targeted delivery; the evidence base here remains predominantly preclinical (cell culture and short-term animal studies), and safety data on long-term nanoparticle exposure in the oral cavity have not been established. Bigels and oleogels, dual-phase systems combining hydrogel and oleogel phases, have been proposed for co-delivering hydrophilic and lipophilic agents together (for example, curcumin with omega-3 fatty acids), but remain at an early formulation-characterization stage [89]. Plant-derived extracellular vesicles, nanoscale structures naturally secreted by plants, have been proposed as a candidate carrier format for bioactive cargo, with preliminary exploratory evidence in periodontitis; this line of work remains at an early, preclinical stage [90].

Despite this innovation, substantial translational barriers remain. Deep pocket penetration and biofilm-associated diffusion barriers continue to limit the effective delivery of even well-engineered formulations to the base of periodontal pockets, where disease activity is often greatest [91]. Regulatory pathways for phyto-nanotechnology and smart biomaterials remain ambiguous in most jurisdictions, with no harmonized framework specifying the evidentiary standard, analogous to a drug, a device, or a combination product, that such formulations must meet before clinical adoption, a gap that a recent review of smart nanogel systems for periodontitis management identified as a rate-limiting step for the entire field rather than a peripheral administrative concern [92]. Pharmacokinetic and pharmacodynamic (PK/PD) characterization of these advanced delivery systems in humans is correspondingly immature: most published work reports in vitro release kinetics or short-term animal pharmacokinetics, and clinical PK/PD data connecting local drug concentration in the periodontal pocket to systemic or even local pharmacodynamic effect over realistic maintenance-therapy timescales are largely absent from the literature reviewed for this manuscript.

Current translational limitations extend beyond engineering design. Most platforms lack large, long-term clinical trials, human pharmacokinetic and pharmacodynamic data, validated manufacturing specifications, and a clear regulatory pathway. Delivery technology may improve local retention, but it does not solve uncertainty about the active agent and may add carrier-related failure modes. Development should proceed only when both components can be standardized and their separate contributions can be tested.

## 9. Clinical Evidence, Safety, and Limitations

A recurring risk in reviews of natural bioactive agents is the implicit conflation of in vitro, animal, and human evidence into a single undifferentiated “evidence base.” This section deliberately separates these tiers because the strength of the periodontal–cardiovascular argument depends critically on which tier a given claim actually rests upon.

At the in vitro level, curcumin, resveratrol, catechins, propolis constituents, and several flavonoids have been reported to inhibit inflammatory signaling, disrupt biofilms, or activate antioxidant responses [10]. These studies identify candidate pathways but do not establish clinical mechanism. Concentrations may be supraphysiological, the polymicrobial and immune context is incomplete, and the formulations tested experimentally often differ from those used clinically. No reviewed human trial showed that a periodontal improvement was mediated by engagement of nuclear factor kappa B, NLRP3, Nrf2, or another proposed pathway.

Animal studies, principally ligature-induced periodontitis models in rodents, add tissue-level and occasionally integrated periodontal-vascular readouts. Ligature placement promotes microbial accumulation and inflammation, so the model is not purely mechanical. Nevertheless, it produces an acute perturbation that differs from chronic human periodontitis in timescale, anatomy, immune adaptation, comorbidity, and treatment context. The grape-seed study measured periodontal and atherosclerotic outcomes in the same model [60], but most animal studies measured local bone loss and cytokines without a paired vascular outcome. These findings support mechanistic plausibility only.

Randomized controlled trials provide the strongest available clinical evidence, but they remain small, short, and formulation specific. Meta-analyses suggest modest changes in selected periodontal surrogates for curcumin [50,51], green tea [55], propolis [56], resveratrol [53], omega-3 fatty acids [69], melatonin [70], and hyaluronic acid [72]. Direct comparisons do not establish equivalence to chlorhexidine and may favor chlorhexidine for some outcomes [52]. None of these trials was designed or powered for myocardial infarction, stroke, cardiovascular mortality, or another cardiovascular event. The route-specific human periodontal evidence, principal methodological limitations, and absence of cardiovascular outcome data are summarized in Table 2.

Beyond individual trials, pooled analyses converge more clearly on limitations than on a reproducible magnitude of benefit. Samples are small, follow-up rarely exceeds six to twelve months, formulations and doses differ, and outcome reporting is inconsistent [93,94]. Trials are geographically concentrated, particularly in parts of Asia. When a field contains many small, single-center studies and negative or null results are less likely to be published, regional and small-study publication bias may inflate pooled benefits and reduce generalizability. Formal assessments were sparse or underpowered, so the direction and magnitude of this bias remain uncertain.

Four recurrent limitations should be considered. First, therapeutic effects have been assessed almost exclusively with surrogate periodontal parameters rather than tooth survival, surgery, vascular outcomes, or cardiovascular events. Second, a single reassuring safety statement cannot be applied across plant metabolites, hormones, fatty acids, allergens, biopolymers, and multicomponent extracts. Short trials cannot exclude rare, delayed, dose-related, or interaction-mediated harms. Third, risk-of-bias reporting, allocation concealment, assessor blinding, and formulation reporting are inconsistent. Fourth, distinctive taste, odor, color, or staining may compromise masking. Together, these limitations reduce confidence in the magnitude, durability, and transportability of reported effects.

Safety considerations also vary according to the route of administration. Systemic supplements can alter drug metabolism, transport, platelet function, blood pressure, glycemic control, or calcium homeostasis independently of periodontal effects. Local products may reduce systemic exposure, but swallowed dose, mucosal absorption, excipients, cross-linkers, and allergy still require study. Absence of interaction data for a local formulation is not evidence of no interaction.

## 10. Research Gaps and a Decision-Gated Translational Roadmap

**Figure 3:**
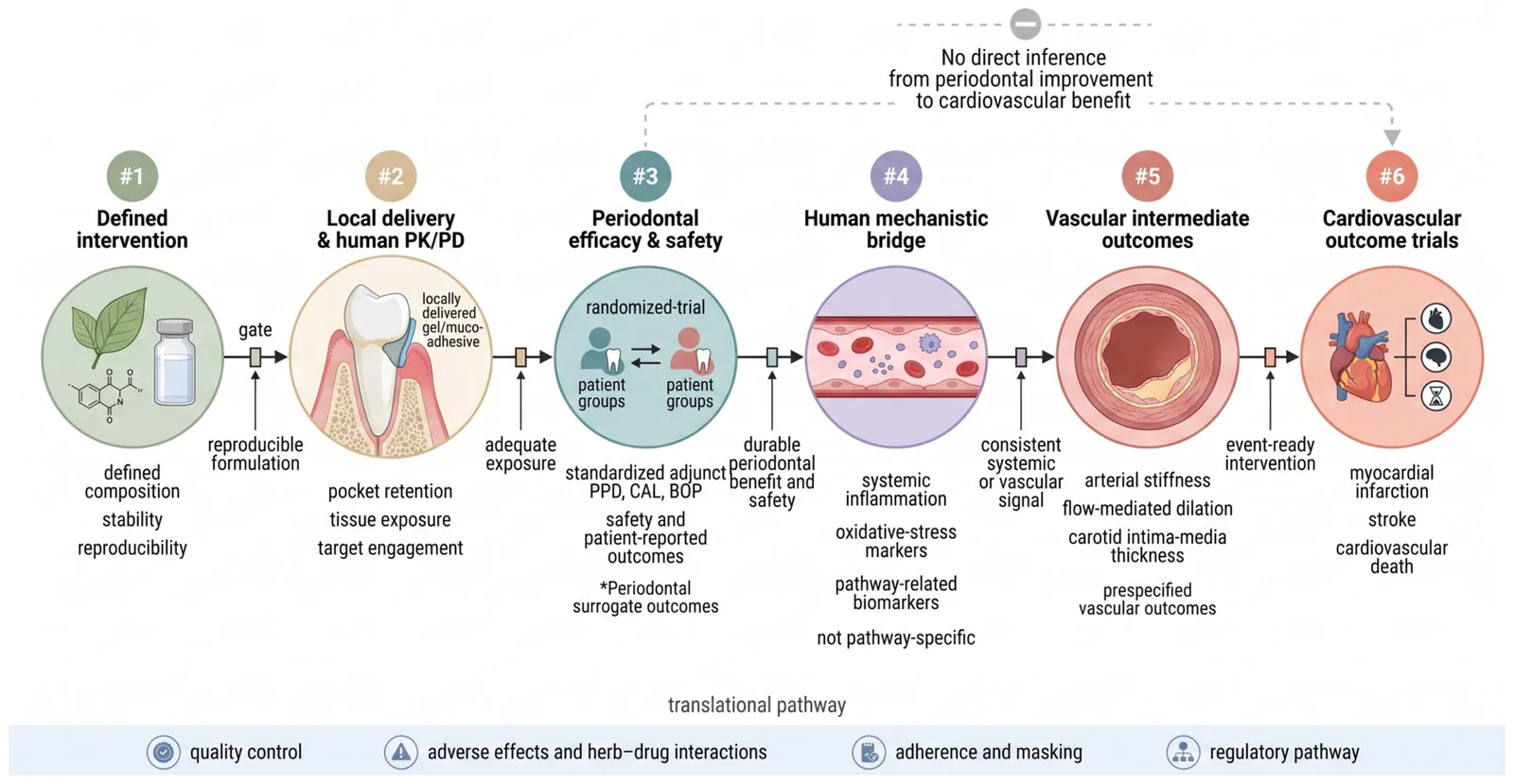
Decision-gated framework for testing cardiovascular relevance of natural periodontal adjuncts. Standardized agent selection is followed by route-appropriate formulation, exposure, safety, and periodontal efficacy testing. Local carriers require independent validation, whereas systemic candidates require separation of direct extra-periodontal effects from effects mediated by periodontal change. Intermediate biomarkers advance a candidate only when they provide prespecified evidence of target engagement, dose response, or mediation; they do not substitute for cardiovascular events. Progression should stop or redirect after inconsistent, non-specific, or unsafe findings. Event-driven trials are justified only after these gates are met [11,49,84,85,86,87,88,89,90,91,92,93,94,95,96]. Abbreviations: BOP, bleeding on probing; CAL, clinical attachment level; PK/PD, pharmacokinetic/pharmacodynamic; PPD, probing pocket depth.

Across the reviewed literature, mechanistic investigation substantially exceeds pharmaceutical and clinical validation. The proposed framework is therefore a series of decision gates, not a guaranteed bridge to cardiovascular trials. A candidate should advance only after its active composition and route-specific formulation are reproducible, exposure and safety are characterized, and a clinically credible periodontal effect is demonstrated. For locally delivered agents, the carrier requires independent validation. For systemic agents, direct cardiovascular effects must be distinguished from effects mediated by periodontal improvement. The proposed decision-gated translational framework is summarized in Figure 3.

Proceeding directly to a cardiovascular-outcome trial could produce an uninterpretable result. Intermediate biomarkers are useful only as prespecified tests of target engagement, dose response, and mediation. A negative, inconsistent, or non-specific signal should halt or redirect development rather than be treated as a weak substitute for a cardiovascular endpoint. Even a favorable vascular surrogate cannot establish prevention of myocardial infarction or stroke.

Safety and pharmacokinetic characterization should be incorporated throughout development. Optimal dosing, long-term adverse effects, formulation-specific toxicity, and drug interactions remain incompletely investigated. These issues are particularly relevant in patients receiving antiplatelet agents, anticoagulants, antihypertensives, glucose-lowering therapies, or lipid-lowering drugs. Table 3 summarizes agent-specific signals and the limits of available evidence [96,97,98,99,100,101,102,103,104,105,106,107,108,109,110,111,112,113].

Regulatory and quality-control limitations also remain substantial. Approval requirements vary across jurisdictions, while botanical preparations may exhibit considerable batch-to-batch variability. For many natural products, agreed chemical fingerprinting methods, purity specifications, stability criteria, and manufacturing standards remain unavailable, limiting the reproducibility required for clinical translation [49].

Prospective trial registration and complete reporting of agent composition, extraction and manufacturing procedures, dose, delivery system, comparator, adherence, masking feasibility, and adverse events would improve transparency, reproducibility, and cross-study comparability.

Current evidence supports selected natural bioactive agents as investigational periodontal adjuncts associated with small, short-term changes in selected surrogate outcomes. Their cardiovascular relevance remains unproven and warrants further investigation through a structured, decision-gated translational approach.

## 11. Conclusions

This review supports three principal conclusions. First, selected natural bioactive agents have been associated with small, short-term improvements in some surrogate periodontal outcomes. The available randomized trials are generally small, short, heterogeneous, and formulation specific. No evidence establishes therapeutic equivalence or non-inferiority to chlorhexidine. Safety and tolerability must be interpreted by agent, dose, formulation, and route rather than generalized across the category.

Second, cardiovascular benefit has not been demonstrated. Periodontitis is consistently associated with atherosclerotic cardiovascular disease in observational studies, but whether this relationship reflects a direct contribution of periodontal inflammation, shared risk factors, or both remains unresolved. The pathways proposed to link natural bioactive agents with vascular biology—including NF-κB, the NLRP3 inflammasome, the Nrf2/HO-1 antioxidant program, RANKL/OPG-dependent bone remodeling, and endothelial nitric oxide signaling—have largely been investigated in separate periodontal and vascular models. No clinical trial identified in this review evaluated cardiovascular events as an outcome.

Third, further progress requires standardized agents and formulations, human pharmacokinetic and pharmacodynamic characterization, adequately powered periodontal trials, and sequential evaluation of systemic and vascular effects before cardiovascular-outcome trials can be justified. Current interpretation is constrained by variable composition, dosing, delivery systems, systemic bioavailability, local retention, and periodontal tissue exposure. With current evidence, natural bioactive agents should therefore be regarded as investigational adjuncts to scaling and root planing rather than substitutes for established mechanical periodontal therapy. 

## Figures and Tables

**Table 1:** Shared pathways, evidentiary level, and current translational ceiling.

Candidate Pathway	Periodontal Evidence	Vascular Evidence	Natural-Agent Evidence	Human Target Engagement	Current Ceiling
NF-κB and inflammatory transcription	Human lesions and biomarkers are compatible with activation; causal inhibition is mainly preclinical [14,74]	Endothelial activation and leukocyte recruitment are supported by separate cardiovascular and pathogen-exposure models [75]	Curcumin, resveratrol, catechins, and flavonoids, mainly in vitro or in animals	Not established in gingival or vascular tissue; CRP and IL-6 are nonspecific	Periodontal surrogates for selected agents; no vascular or cardiovascular-event benefit
NLRP3, IL-1β/IL-18, and pyroptosis	Periodontal observations and experimental inhibition in animal models [17,18,64]	Experimental atherosclerosis and macrophage-pyroptosis literature [76]	Ursolic acid and related candidates have preclinical activity [64]	No trial confirmed inflammasome assembly, caspase-1 inhibition, or reduced pyroptosis	Preclinical mechanistic efficacy only
Nrf2/KEAP1 and redox control	Cell and animal evidence for oxidative-stress modulation [77]	Endothelial and pathogen-exposure models support Nrf2/eNOS plausibility [66,78]	Curcumin, resveratrol, catechins, and cinnamaldehyde have experimental evidence	Not established for the clinical periodontal formulations	Periodontal surrogates for some agents; vascular experimental outcomes only
Nitrate–nitrite–nitric oxide pathway	Small human study showed impaired ex vivo nitrate reduction and improvement after treatment [39,40]	Systemic nitric oxide relevance is physiologically plausible	No natural periodontal adjunct has validated this pathway	Not established; basal plasma nitrate and nitrite did not change	Human oral functional observation only
RANKL/OPG and bone remodeling	Strong periodontal rationale, with human association and preclinical intervention data	Separate vascular-calcification and endothelial-injury literature [79]	Polyphenols and terpenoid-related candidates are mainly preclinical; vitamin D evidence concerns deficiency correction	No convincing demonstration that pathway change mediates a human clinical outcome	Periodontal surrogates and preclinical bone outcomes
Inflammation resolution and efferocytosis	Resolution biology is relevant to periodontal inflammation	Resolution pathways influence vascular inflammation in separate literature	Omega-3 provides systemic substrate for pro-resolving mediators; hinokitiol and dipotassium glycyrrhizate enhance efferocytosis in vitro [65]	Not routinely measured in periodontal trials	Small periodontal surrogate effects for omega-3; no periodontal-mediated vascular effect
Biofilm-related activity	In vitro inhibition is reported for catechins, curcumin, propolis, and extracts [54]	Cardiovascular relevance would require reduced dissemination or systemic exposure	Concentration- and formulation-dependent in vitro evidence	Sustained subgingival exposure and ecological engagement are rarely shown	Short-term periodontal outcomes; no vascular or event evidence

Abbreviations: BOP, bleeding on probing; CRP, C-reactive protein; eNOS, endothelial nitric oxide synthase; IL, interleukin; KEAP1, Kelch-like ECH-associated protein 1; NLRP3, NOD-like receptor family pyrin domain containing 3; OPG, osteoprotegerin; RANKL, receptor activator of nuclear factor kappa-B ligand.

**Table 2:** Route-specific human periodontal evidence and cardiovascular outcome boundaries.

Agent or Preparation	Route and Comparison	Evidence Base	Verified Periodontal Estimate	Principal Limitation	Cardiovascular Outcome
Curcumin or turmeric-derived formulations	Site-directed local + SRP vs SRP alone	23-trial meta-analysis [50]	PPD MD −0.47 mm (95% CI -0.88 to −0.06; I^2^ = 95.5%); CAL MD -0.33 mm (95% CI -0.54 to -0.11; I^2^ = 62.3%)	Small effect, high heterogeneity, variable formulations	None
Curcumin vs chlorhexidine	Primarily local or topical active comparisons	26 RCTs, 1266 participants [52]	Chlorhexidine favored for PPD, SMD 0.883 (95% CI 0.419 to 1.347), and attachment loss, SMD 0.539 (95% CI 0.019 to 1.058)	24 RCTs at high risk of bias; no non-inferiority or equivalence design	None
Green tea catechins	Site-directed local + SRP vs SRP alone	4-trial meta-analysis [55]	Additional PPD reduction 0.74 mm (95% CI 0.35 to 1.13)	Few small trials; routes and formulations differ	None
Propolis	Subgingival + NSPT vs NSPT alone or placebo	6 studies, 3 pooled; 30-90 days [56]	PPD reduction 1.49 mm at 30–45 days and 0.80 mm at 90 days; CIs unavailable in indexed abstract	High risk of bias, very-low-certainty evidence, allergy	None
Mixed local natural products	Subgingival + SRP vs SRP alone or placebo	27 trials; meta-analysis at 3-6 months [93]	PPD reduction 0.72 mm (95% CI 0.23 to 1.22); CAL gain 1.07 mm (95% CI 0.36 to 1.78)	I^2^ > 69%; products not interchangeable	None
Grape-seed extract	Site-directed gel + SRP	Two RCTs: 86 sites in 5 patients and 72 randomized patients [58,59]	No PPD benefit in the site-level study; PPD and relative attachment favored adjunct in the patient-level study, but no indexed effect size or CI	Discordant designs and results; no pooled estimate	Animal evidence only [60]
Hyaluronic acid	Local reparative gel + NSPT vs NSPT alone	23-study review; 12 pooled [72]	PPD WMD -0.46 mm (95% −0.89 −0.04); CAL WMD −0.35 mm (95% CI −0.61 to -0.09); BOP not significant	Prediction intervals crossed null; formulation heterogeneity	None
Topical herbal rinses or dentifrices	Mouth rinse or dentifrice vs placebo, mechanical care, or CHX	Mostly short gingivitis studies [44,94]	No reliable periodontitis-specific pooled PPD or CAL estimate	Limited subgingival exposure; no equivalence designs	None
Resveratrol	Systemic or topical + NSPT	7 RCTs, 402 participants [53]	PPD MD -0.42 mm (95% CI -0.59 to −0.25; I^2^ = 99%); CAL MD −0.36 mm (95% CI −0.52 to −0.19; I^2^ = 98%)	Extreme heterogeneity; no standardized regimen	None
Omega-3 fatty acids	Systemic supplementation + SRP	16 RCTs; 9 pooled [69]	At 3 months, CAL MD -0.49 mm (95% CI −0.75 to −0.23; I^2^ = 84%) and PPD MD -0.44 mm (95% CI −0.62 to -0.25; I^2^ = 81%)	Variable EPA/DHA exposure, aspirin cointervention, dose undefined	None in periodontal trials; direct systemic effects possible
Melatonin	Systemic or local + NSPT	18 RCTs, 828 participants [70]	At 3 months, CAL −0.41 mm (95% CI −0.69 to −0.13) and PPD −0.29 mm (95% CI −0.53 to −0.05); BOP not significant	Prediction intervals crossed null; mixed routes	None
Vitamin D	Systemic deficiency correction + SRP	Pilot RCT, 27 randomized [71]	No significant between-group periodontal difference	Deficient population; no inference for vitamin-D-replete patients	None
Isoflavones, isolated alkaloids, individual terpenoid-related compounds, or isolated natural peptides	Candidate local or systemic agents	No adequate dedicated adjunctive evidence	No verified pooled PPD, CAL, or BOP estimate	Predominantly preclinical	None

Abbreviations: BOP, bleeding on probing; CAL, clinical attachment level; CHX, chlorhexidine; CI, confidence interval; DHA, docosahexaenoic acid; EPA, eicosapentaenoic acid; MD, mean difference; NSPT, nonsurgical periodontal therapy; PPD, probing pocket depth; RCT, randomized controlled trial; SMD, standardized mean difference; SRP, scaling and root planing; WMD, weighted mean difference. Negative MD values favor the adjunct when the outcome is expressed as residual depth or loss; positive reductions or gains are reported as stated by the source.

**Table 3:** Agent-specific safety and interaction considerations relevant to cardiovascular medication use.

Agent Or class	Route or Evidence context	Human Safety or Interaction Signal	Interpretation for Periodontal Research
Curcumin or turmeric	Mainly systemic high-dose evidence; local exposure uncertain	Human probe studies and platelet-related data indicate formulation-specific interaction potential; enhanced-bioavailability products have a liver-injury signal [97,98,99]	Record formulation and bioenhancers; review anticoagulants and liver risk; measure systemic exposure when relevant
Resveratrol	Systemic, 1 g/day human probe study	CYP3A4, CYP2D6, and CYP2C9 probe disposition changed [100]	Review relevant cardiovascular drug substrates; clinical outcome evidence is absent
Green tea or EGCG	Brewed tea and concentrated systemic products	Nadolol exposure fell substantially; rosuvastatin exposure changed in small studies; a warfarin interaction is case-based [101,102,103]	Separate tea, extract, and local formulations; document beta-blockers, statins, and anticoagulants
Propolis	Local oral products; one oral standardized-extract probe study	Product-specific metabolism findings have limited generalizability; contact allergy is established [104,105]	Screen bee-product allergy and report local reactions prospectively
Berberine	Systemic repeat-dose evidence	Human evidence shows inhibition of CYP2D6 and CYP2C9 [106]	Medication review is required; local and systemic exposure cannot be conflated
Essential oils and terpenoid-related products	Mainly local products	Mouthrinses can cause irritation, mucosal lesions, and taste disturbance [107]	Report exact composition, concentration, vehicle, swallowed dose, and local tolerability
Omega-3 fatty acids	Systemic supplements and prescription products	No overall bleeding increase in pooled trials; high-dose purified EPA had a small absolute bleeding excess [108]	Distinguish formulation and dose; review antithrombotic therapy and individual bleeding risk
Melatonin	Systemic	A nifedipine-specific blood-pressure interaction and altered insulin sensitivity have been reported [109,110]	Record antihypertensive and diabetes therapy, dose, timing, and formulation
Vitamin D	Systemic deficiency correction	Hypercalcemia risk with thiazides in susceptible patients [111]	Distinguish deficiency treatment from pharmacological supplementation; monitor calcium when indicated
Hyaluronic acid	Local reparative biomaterial	Few reported local events, but follow-up and systemic exposure data are limited [112]	Report source, cross-linkers, vehicle, local reactions, and adverse-event ascertainment
Polyherbal mixtures	Local or systemic, often incompletely characterized	Safety and interaction risk cannot be assigned when identity, dose, or contaminants are uncertain [113]	Require botanical identity, extraction method, batch testing, route, dose, and concomitant medication

Abbreviations: CYP, cytochrome P450; EGCG, epigallocatechin-3-gallate; EPA, eicosapentaenoic acid. Signals are formulation and route specific and should not be generalized to the entire umbrella category.

## Data Availability

No new datasets were generated or analyzed in this study. All data discussed in this review were obtained from previously published studies cited in the article. The literature was retrieved from publicly accessible databases, including PubMed/MEDLINE (https://pubmed.ncbi.nlm.nih.gov/; accessed on 4 June 2026) and Europe PMC (https://europepmc.org/; accessed on 4 June 2026).
